# Risk Levels of Toxic Cyanobacteria in Portuguese Recreational Freshwaters

**DOI:** 10.3390/toxins9100327

**Published:** 2017-10-18

**Authors:** Carina Menezes, Catarina Churro, Elsa Dias

**Affiliations:** Department of Environmental Health, National Institute of Health Dr. Ricardo Jorge, Av. Padre Cruz, 1649-016 Lisboa, Portugal; carina.menezes@insa.min-saude.pt (C.M.); catarina.churro@gmail.com (C.C.)

**Keywords:** cyanobacteria, cyanotoxins, microcystins, recreational freshwaters, guidelines, risk levels

## Abstract

Portuguese freshwater reservoirs are important socio-economic resources, namely for recreational use. National legislation concerning bathing waters does not include mandatory levels or guidelines for cyanobacteria and cyanotoxins. This is an issue of concern since cyanotoxin-based evidence is insufficient to change the law, and the collection of scientific evidence has been hampered by the lack of regulatory levels for cyanotoxins in bathing waters. In this work, we evaluate the profile of cyanobacteria and microcystins (MC) in eight freshwater reservoirs from the center of Portugal, used for bathing/recreation, in order to determine the risk levels concerning toxic cyanobacteria occurrence. Three of the reservoirs did not pose a risk of MC contamination. However, two reservoirs presented a high risk in 7% of the samples according to the World Health Organization (WHO) guidelines for MC in bathing waters (above 20 µg/L). In the remaining three reservoirs, the risk concerning microcystins occurrence was low. However, they exhibited recurrent blooms and persistent contamination with MC up to 4 µg/L. Thus, the risk of exposure to MC and potential acute and/or chronic health outcomes should not be disregarded in these reservoirs. These results contribute to characterize the cyanobacterial blooms profile and to map the risk of toxic cyanobacteria and microcystins occurrence in Portuguese inland waters.

## 1. Introduction

Cyanobacterial blooms are becoming more frequent and widespread in freshwater reservoirs worldwide, mainly due to anthropogenic activities and to climate changes [1]. Some toxic cyanobacterial species are invading and expanding to new geographic regions [1,2]. Considering the ability of cyanobacteria to produce several types of cyanotoxins, the monitoring of freshwater reservoirs is crucial to prevent acute and chronic health effects [1,3]. This is particularly important in those reservoirs used as a source of drinking water and for recreational activities, where people are exposed to cyanotoxins by oral, dermal and inhalation routes [1].

Portuguese surface freshwater resources are largely used for energy production, irrigation, recreational activities and as a source of drinking water for human consumption [4]. An important proportion of those resources are river-derived artificial reservoirs, such as water dams. These artificial barriers alter the natural river flow and create lentic water bodies that are vulnerable to eutrophication [4], which favors cyanobacteria proliferation [5]. In fact, cyanobacterial blooming in Portuguese surface freshwaters is common and often associated with cyanotoxin production [6,7,8,9,10,11,12,13]. Thus, the monitoring of cyanobacteria and cyanotoxins is a major issue for public health.

The Portuguese legislation for drinking water, Decreto-Lei nº306/2007 [14], transposed from the European Drinking Water Directive [15], established the regulatory level of 1 µg/L for total microcystins in treated water. This parameter is determined when eutrophication of water is suspected and when the number of potentially toxic cyanobacteria exceeds 2000 cells/mL. Conversely, the Portuguese legislation concerning the quality of bathing water [16,17], that was transposed from the European Bathing Water Directive [18], did not include any guideline for cyanobacterial cells or microcystin concentrations. It only recommends that when the bathing water profile indicates a potential for cyanobacterial proliferation, appropriate monitoring should be carried out to enable timely identification of health risks. When cyanobacterial proliferation is detected visually, it is the responsibility of the local health delegate to evaluate the health risk. If any risk has been identified or presumed, the health and environmental authorities should implement the adequate management measures to prevent exposure, including information to the public.

According to national specificities and circumstances, some European countries complemented the European Bathing Water Directive [18] and implemented their own guidance or regulations, based on cyanobacterial cell numbers, biovolumes, pigments and/or cyanotoxin concentrations [19,20].

The lack of a national database compiling the results of cyanobacteria and cyanotoxin monitoring hinders the characterization of Portuguese inland waters concerning toxic cyanobacterial blooms and the associated risks for public health and, in consequence, the implementation of guidelines.

Furthermore, we must take into account that the use of those reservoirs is increasing, not only for the production of drinking water, but also for ludic activities, such as water sports, fishing, sailing, swimming and bathing. In addition, the vicinities of those reservoirs host camping, fairs, national and international summer music festivals, natural parks and hunting reserves. Thus, the potential risks associated with these reservoirs must be evaluated and managed in order to protect the health of their users.

In this work, we compiled the results obtained during 14 years of monitoring of cyanobacteria and microcystins in eight freshwater reservoirs located in the centre of Portugal used for bathing and recreational activities. Based on these data, we profiled cyanobacterial blooms and determined the risk levels of toxic cyanobacteria occurrence and putative human exposure scenarios in these reservoirs.

## 2. Results

### 2.1. Cyanobacteria and Microcystins Profiles in the Studied Reservoirs

**Figure 1:**
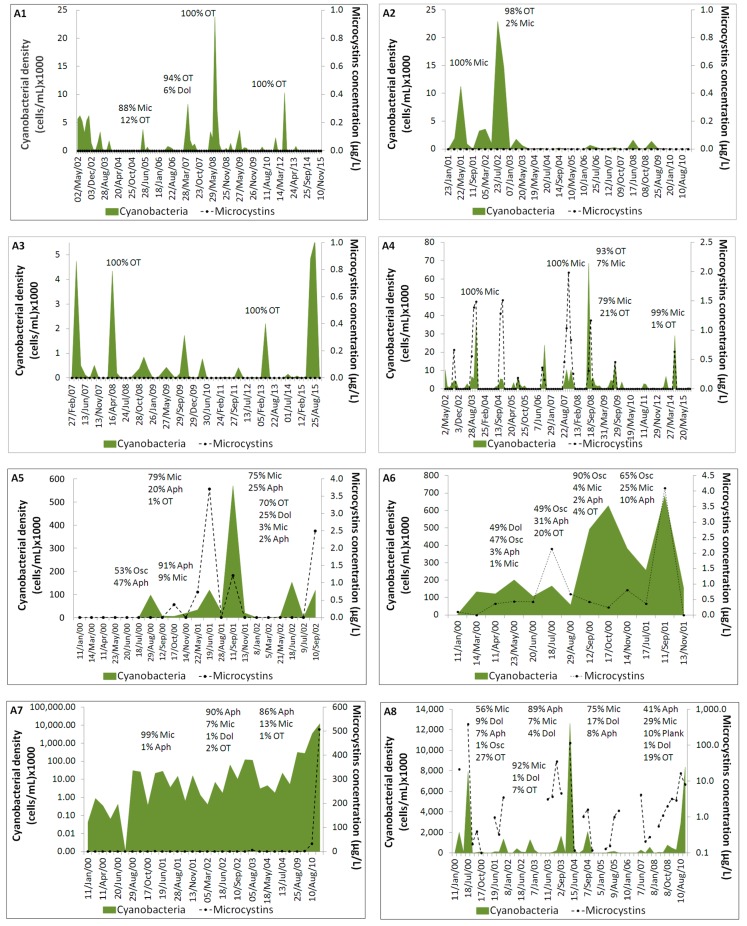
Cyanobacteria densities and microcystin concentrations over the monitoring periods for reservoirs A1, A2, A3, A4, A5, A6, A7 and A8. The arrows indicate the main cyanobacterial genera in the selected time points. Legend: Aph—*Aphanizomenon* spp.; Dol—*Dolichospermum* spp.; Mic—*Microcystis* spp.; Osc—*Oscillatoria* spp.; Plank—*Planktothrix* spp.; OT—Other *taxa*.

The variation of cyanobacterial density and microcystin concentration in the monitored freshwater reservoirs is presented in Figure 1. The percentage of blooms and of microcystin-positive samples in each reservoir is shown in Table 1. The cyanobacteria species profile of each reservoir is indicated in Table 2.

The studied reservoirs exhibited distinct scenarios concerning the variation of cyanobacteria and microcystins. In reservoirs A1, A2 and A3, the cyanobacterial cell densities were mostly below 10,000 cells/mL never exceeding 25,000 cells/mL (Figure 1). The overall bloom frequency was 30%, 25% and 15%, respectively (Table 1). Although potentially toxic cyanobacteria occurred (Table 2), mainly *Microcystis aeruginosa* (16–28% of the samples), their bloom frequency was below 10% and microcystins were never detected in the samples (Figure 1, Table 1).

In reservoir A4, the cyanobacterial density reached up to 80,000 cells/mL (Figure 1) and the bloom frequency was 44% (Table 1). Microcystins were detected in 16% of the samples (Table 1), but never exceeded 2 µg/L (Figure 1). Microcystins peaks coincided with the dominance of *Microcystis* species. Indeed, *M. aeruginosa* was the predominant species (Table 2).

In reservoir A5, the bloom frequency was high (77%) and, in A6, blooms were detected during the entire two-year monitoring period (Figure 1, Table 1). In both reservoirs, cyanobacteria often reached extreme densities (above 100,000 cells/mL) (Figure 1) and potentially toxic species, such as *Aphanizomenon* spp., *Dolichospermum* spp., *Microcystis* spp., *Oscillatoria* spp. and *Cylindrospermopsis raciborskii* (Table 2), were often predominant. Microcystins were detected in 24% of samples from A5 and in 85% of samples from A6 (Table 1), but microcystin concentrations were below 4 µg/L. 

Intense blooms were also detected in reservoirs A7 and A8, with a frequency of 69% and 79%, respectively (Figure 1, Table 1). Cyanobacteria surpassed the level of 10^6^ cells/mL, particularly in A8 (Figure 1). Microcystins occurred in 38% (A7) and 58% (A8) of the samples (Table 1). In reservoir A7, high levels of microcystins were detected in samples from August and October 2010 (35 and 506 µg/L, respectively). In reservoir A8, high microcystin concentrations were also detected in the samples collected in May 2000 (21 µg/L), July 2000 (389 µg/L), August 2003 (35 µg/L) and May 2004 (115 µg/L).

The toxin peaks coincided with the dominance of *Microcystis aeruginosa*, *Dolichospermum* spp., *Aphanizomenon* spp. and/or *Oscillatoria* spp. (Figure 1, Table 2).

The seasonality of blooms in the reservoirs can hardly be compared, considering that the monitoring period was not the same for all the reservoirs. Nevertheless, in general, the period of 2000–2002 and 2007–2009 corresponded to the highest bloom frequencies (Figure 1).

On the other hand, most blooms occurred in spring and summer (April to September). It was the case of reservoirs A1, A2 and A3. However, blooms were also detected in October (A4 in 2003/2004, A6 in 2000, A7 in 2011 and A8 in 2000/2004/2007/2008/2010) and occasionally in November (A4 in 2007/2009, A5/A6 in 2000/2001 and A7 in 2001), December (A4 in 2007) and January (A8 in 2002/2003). Except for A4, where *Microcystis* species were always dominant, those autumn/winter blooms were mostly composed by filamentous species, such as *Aphanizomenon* spp., *Dolichospermum* spp., *Cylindrospermopsis raciborskii*, *Oscillatoria* spp. and *Planktothrix* spp. (Figure 1, Table 2).

### 2.2. Risk Levels of Toxic Cyanobacteria Occurrence in the Studied Reservoirs

**Figure 2:**
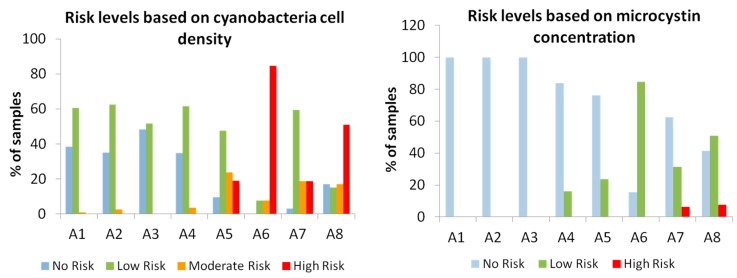
Risk levels of the eight freshwater reservoirs based on cyanobacterial cell density (**a**) and on microcystin concentration (**b**).

The risk levels concerning cyanobacteria density and microcystin concentrations, based on the WHO Guidelines for bathing waters (see Material and Methods section), were determined for each reservoir as shown in Figure 2. According to the cell-based criteria, all reservoirs presented some degree of risk. In reservoirs A1 to A4, the risk based on cell numbers was generally low, although, in reservoirs A1, A2 and A4, a moderate risk was determined in less than 4% of the samples (Figure 2a). Reservoirs A5, A6, A7 and A8 exhibited a high-risk level in 20–80% of the samples (Figure 2a). However, the severity of the risk was reduced in all reservoirs when the microcystin-based criteria was used (Figure 2b), and only reservoirs A7 and A8 showed a high-risk level in approximately 7% of the samples (Figure 2b).

### 2.3. Human Exposure Scenarios to Microcystins during Recreational Activities in the Studied Reservoirs

The minimum water volumes required to reach the tolerable daily intake (TDI) through accidental swallowing in the reservoirs are shown in Table 3. Reservoirs A1, A2 and A3 did not pose a risk of exposure since no microcystins were detected. In reservoirs A4, A5 and A6, the volume of ingested water required to overcome the TDI was approximately 100 mL or higher. Conversely, in some blooms from reservoirs A7 and A8 only small volumes of water would be sufficient to overcome the TDI and constitute a real risk scenario of human acute intoxication with microcystins (Table 3). 

## 3. Discussion

The study of the occurrence of cyanobacteria in Portuguese freshwater reservoirs dates back to the 1930s, but it was only in the 1990s that research on the toxicity and distribution of toxic species gained special emphasis, with the works of Vasconcelos and collaborators, particularly in the north of the country (revised in [21]). In the late 90s, monitoring programs of cyanobacteria and cyanotoxins in surface freshwater reservoirs were implemented by the Portuguese health authorities [7,22]. However, if the quality of drinking water is, to some extent, safeguarded by Portuguese legislation [14], the national legislation concerning bathing waters do not include yet parametric values for cyanobacteria and microcystins. 

With this work, we intended to evaluate the trend of cyanobacterial blooms and microcystins in eight freshwater reservoirs used for recreation and determine the risk levels and putative human exposure scenarios associated with those blooms.

The results show that the studied freshwater reservoirs have distinct profiles of cyanobacteria proliferation and microcystins occurrence and, consequently, different risk scenarios concerning the potential human exposure to cyanotoxins. Three reservoirs (A1, A2 and A3), did not present a potential risk of human exposure to microcystins. Although a low risk based on cyanobacteria density was observed in more than 50% of the samples and despite potentially toxic cyanobacteria occurred, mainly *Microcystis aeruginosa*, microcystins were never detected in water samples. These reservoirs are classified as mesotrophic (A1 and A3) and oligotrophic (A2), which might explain the low cyanobacteria density along all the monitoring period (from 9 to 14 years). Besides, non-toxic *Microcystis aeruginosa* strains might have been predominant within the cyanobacterial communities in these reservoirs. In three reservoirs (A4, A5 and A6), the occurrence of microcystins represented a low risk of potential acute effects because toxin levels were always below 4 μg/L and the water volumes required to produce such effects in adults were quite improbable. However an accidental swallowing of 100 mL of water in reservoirs A5 and A6 could already represent a health risk in children weighing up to 10 Kg. In addition, the risks resulting from repeated exposures should not be disregarded, particularly in the eutrophic reservoir A6, where the risk level based on cyanobacteria density was high and where blooms were quite persistent. Potential toxic genus (*Aphanizomenon*, *Cylindrospermopsis*, *Dolichospermum*, *Microcystis*, *Oscillatoria*, *Planktothrix*) were dominant and, inclusively, two strains of *M. aeruginosa* isolated from reservoir A4 in October 2003 and from reservoir A5 in September 2002 were previously reported as producers of several microcystin variants (MCLR, MCRR and MCYR) [23,24,25]. Thus, the occurrence of microcystins at those time points was, at least, attributed to the species *M. aeruginosa*. Although no cyanobacterial strains were isolated from reservoir A6 during the monitoring period, this reservoir has a previous history concerning the occurrence of toxic strains of *M. aeruginosa*, producers of several variants of microcystins [23,24,25]. In two reservoirs (A7 and A8), the high risk level of 20 µg MCLR/L established by WHO [26] was exceeded in 7% of the samples, reaching a maximum of 506 µg/L in reservoir A7 and 389 µg/L in reservoir A8. However, the reality of these two reservoirs was quite different. The highest concentrations of microcystins in the reservoir A7 were detected only in the period of August–October 2010, remaining at residual levels (bellow 1 µg/L) in the other periods. In reservoir A8, microcystins peaked in distinct years and persisted along the monitoring period at residual levels or between 1 µg/L and 20 µg/L. In addition, the risk level based on cyanobacterial density was in general low at reservoir A7, whereas a high-risk level was determined in 51% of the samples from reservoir A8. In both reservoirs, realistic microcystins exposure scenarios were identified, more often in reservoir A8. Indeed, in some toxic bloom occurrences, only very small volumes of ingested water (1–5 mL) would be required to induce harmful health effects. Despite the great diversity of cyanobacteria in these reservoirs, the production of microcystins has only been confirmed to date in *M. aeruginosa* strains isolated from reservoir A8 [23,24,25]. 

Overall, we found a great diversity of species, being the most prevalent, among the potential toxic genera, *Microcystis*, *Dolichospermum, Aphanizomenon* and *Oscillatoria*. Previously, other authors also reported the prevalence and dominance of *Microcystis* spp. in freshwaters from the north [27,28] and south [10,23,24] of Portugal. It should be noted, however, that blooms of *Dolichospermum* (formerly named as *Anabaena*) [10,23,28], *Aphanizomenon* [29,30] and *Planktothrix* [12,23,31,32] have also been reported in Portuguese freshwaters. Other studies showed that the MCLR variant is the most common, with a proportion of 44 to 100% in relation to the total microcystins content [6,25,33]. It was also concluded that MCRR, MCYR, MCAR and [D-Asp3] MCLR also occurs, but in a lower proportion suggesting that the methylated variants of microcystins are the most common in Portugal [6,25,33]. Additionally, other cyanotoxins have also been detected in blooms or in cultured strains isolated from natural samples, such as saxitoxins [8,9,29,34,35] and anatoxin [36]. Besides, if it was once considered that some cyanobacteria species were confined to certain geographical locations, namely tropical regions, nowadays those species and their toxic compounds are emerging and spreading into other regions. It is the case, for example, of *Cylindrospermopsis raciborskii* [2,37] and the amino acid *β*-*N*-methylamino-l-alanine (BMAA) [38] that have been detected in reservoirs and/or estuaries in several European countries, including Portugal. In reservoirs A6 and A8, *C. raciborskii* also occurred sporadically, but its toxicity was not evaluated. This points out that the risk levels in the studied reservoirs may be underestimated considering that cyanobacterial species that are potentially producers of other toxins also occur frequently and at high cell densities.

We should also emphasize that, besides the acute cases of illness in humans exposed to microcystins during recreational activities [39,40], continuous exposure to water contaminated with low concentrations of microcystins may also pose a risk of chronic health effects [1,3,41]. Thus, the health risks resulting from human and animal exposure to cyanobacterial toxins should not be disregarded in five of the eight evaluated recreational freshwater reservoirs (A4 to A8). Indeed, although recreational exposure is intermittent by nature, the bathing season in this region may start in early spring and last until early autumn. Additionally, other activities besides bathing, such as water sports, occur along the year. Furthermore, exposure routes other than ingestion should be considered, such as aerosol exposure to low concentrations of microcystins (2 to 5 μg/L) during recreational activities [42,43].

On the other hand, it has been reported that cyanotoxins may accumulate in horticultural crops [44] that might have negative consequences to the physiology of the plants as well as to the health of the consumers. Thus, potential economic loses and human/animal health risks should also be considered in Portuguese surface freshwater reservoirs, since many are also used for irrigation, such as reservoirs A5, A7 and A8.

The available epidemiologic data concerning human exposure to cyanotoxins in Portuguese freshwaters is restricted to two situations. The first occurred in 1993 in a dialysis unit in Évora Hospital (Alentejo, south Portugal), where twenty dialysis patients died [45,46]. This was an episode very similar to the well-known Caruaru incident in Brazil. The water that supplied the city was heavily contaminated with *Microcystis*, *Aphanizomenon* and *Dolichospermum*, but it also contained high levels of aluminum [45,46]. Unfortunately, cyanotoxins analyses were not performed in the victims or in the water, and the correlation with cyanobacteria remained uncovered. However, an epidemiological study of ecological type showed a correlation between the occurrence of cyanobacteria and the change in liver enzymes in the population supplied by the contaminated water reservoir. This was the first time that the occurrence of cyanobacteria was considered as a risk factor to human health in Portugal [45]. A more recent study from Bellém et al. [13] alerted for the potential public health concern in six freshwater reservoirs from Alentejo (Alvito, Boavista, Enxoé, Roxo, Monte Novo, Vigia), considering the dominance of potentially toxic cyanobacteria from spring to late autumn, with some recorded cell density values corresponding to medium/high risk factor, according to the guidelines from WHO [26]. Unfortunately, this paper does not mention the levels of cyanotoxins in those reservoirs. Nevertheless, the same author developed a historical prospective study (2000–2010), in which two populations from Alentejo [one supplied by freshwater reservoirs contaminated with cyanobacterial blooms (exposed population) and one supplied by other water sources (unexposed population)] were compared relatively to the laboratory indicators of hepatic pathology. According to the author, the exposure to cyanotoxins had an impact on liver disease, attested by the high values of transaminases and the higher incidence of hepatocarcinoma in the exposed population [47]. There is no national data on adverse health effects resulting from the exposure to cyanotoxins during recreational activities, but those previous reports point out the potential risk associated with surface freshwaters reservoirs from south Portugal. These regions have a Mediterranean weather, with mild winters and hot and dry summers, being especially vulnerable to climate changes, and severe or extreme drought in particular, which may favor eutrophication and cyanobacteria blooming [5,48,49,50].

It was not our goal with the present paper to draw conclusions about the causes of the blooms in the studied reservoirs. Indeed, the monitoring period was not the same among them and, consequently, the seasonality of blooms in the reservoirs can hardly be compared. Nevertheless, we observed that, in a general way, the periods of 2000–2002 and 2007–2009 corresponded to the highest bloom frequencies. It is interesting to note that these dates were preceded by periods of extreme droughts, namely in 1998–1999 and 2004–2006 [51]; the latest, inclusively, was the most extreme drought in terms of territorial extension of the last 65 years. We can hypothesize that these climatic extremes may have provided the conjugation of biotic and/or abiotic factors that, in turn, triggered the increase of blooms in subsequent years.

On the other hand, although the blooms were detected mainly in spring and summer seasons, the reservoirs A4 to A8 also presented blooms in autumn and winter. These blooms occurred when the average air temperature was above normal for that period (e.g., January 2003, November/December 2007, November 2009) or in rainy months that were preceded by heat waves and/or dry summers (e.g., October 2003, October 2011) [51,52]. This is similar to what has been previously described in Mediterranean areas, such as Greece, where the blooms may last until December or even persist throughout the year [49]. Most of those blooms were composed by filamentous species, namely heterocysts and akinetes forming species, such as *Aphanizomenon* spp., *Dolichospermum* spp. and even *Cylindrospermopsis raciborskii*, probably due to their highly successful adaptive strategies.

Many countries that regulate cyanotoxins in drinking water use a parametric value based on the WHO Guideline Value for MCLR of 1 μg/L [20]. However, and according to regional specificities, some countries also adopted guidance/alert/maximum levels for cylindrospermopsin (Australia, New Zealand, Brazil), saxitoxin (Australia, New Zealand, Brazil) and anatoxin-a (Canada, New Zealand) [19,20]. In what concerns recreational waters, most countries use guidance values based on cyanobacterial biomass (cell density, chlorophyll-a, biovolume) reflecting, indirectly, potential hazardous microcystin concentrations [19,20]. The risk of health problems due to the presence of other cyanotoxins in bathing waters is recognized, but the adoption of guidance values for those cyanotoxins in water bodies used for recreation is still an issue under discussion [19].

## 4. Conclusions

In summary, we identified potential risk scenarios of human exposure to cyanobacteria and microcystins in five of the eight monitored recreational freshwater reservoirs located in central Portugal. Considering the cyanobacterial diversity observed in those reservoirs, we hypothesize that other cyanotoxins might occur and, consequently, that these risks might be underestimated. Therefore, the inclusion of other cyanotoxins in monitoring plans will be essential to clearly identify the risks associated with cyanobacteria in such reservoirs, particularly, the risks arising from exposure during recreational activities. Additionally, the systematization of monitoring data at a national scale would enable to obtain scientific-based evidence that can contribute to establish regulatory or guideline values for cyanobacteria and cyanotoxins in recreational freshwaters and, thus, to prevent health risks in such important socio-environmental water resources.

## 5. Materials and Methods

### 5.1. Study Approach

For this study, a retrospective approach was used in which data of cyanobacteria and microcystins obtained between the years 2000 and 2015 was compiled. These analyses were performed in our laboratory, in the scope of the water quality monitoring programs implemented by the local water management entities. The sampling plan and the collection of water samples were the sole responsibility of the local authorities. Notice that, due to confidential obligations, we cannot identify the reservoirs. 

### 5.2. Sampling and Sample Processing

**Figure 3:**
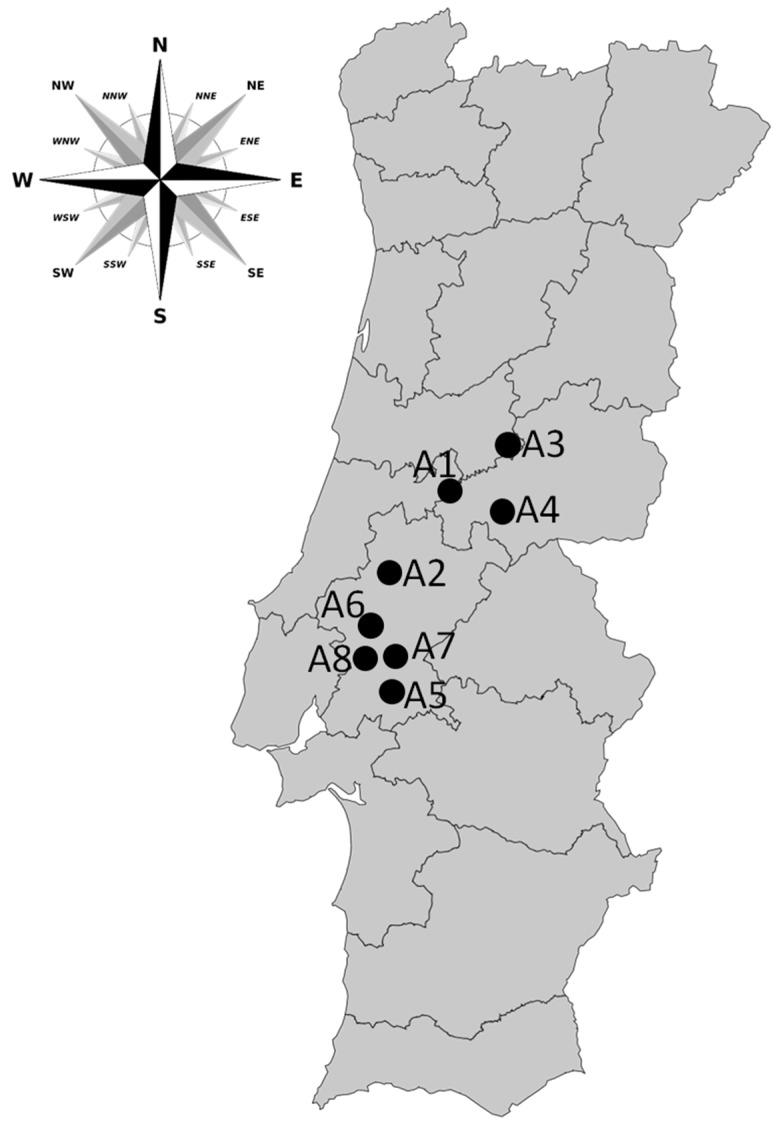
Geographic locations of the reservoirs studied in Portugal.

Sampling was performed in eight freshwater reservoirs located in the center of Portugal (Figure 3), between 2000 and 2015. Depending on the reservoir, the monitoring period varied between 2 and 14 years (Table 4). Sampling frequency varied between one week to three months, depending on the cyanobacterial cell density and on the microcystin concentration detected. Samples of 1 L were collected from the water surface (<1 m depth) and transported to the laboratory under refrigerated conditions.

Aliquots of 100 mL were preserved with Lugol’s solution (1 drop/10 mL) for phytoplankton identification and quantification. Two aliquots of 5 mL were kept frozen (−20 °C) until microcystin analysis. 

### 5.3. Phytoplankton Analysis

Phytoplankton species were analyzed according to the Utermöhl technique [55]. Briefly, previously preserved 100 mL aliquots were homogenized by inversion and an adequate volume (5 to 25 mL, depending on sample density) was used to fill a sedimentation chamber. Chambers were left to settle for 24 h, after which observation was performed by phase contrast optical microscopy (Olympus CK40). Taxonomic identification was based on microscopic observation of distinctive morphological features and identification keys according to annex D from reference [55]. A sample was considered to have a bloom whenever the cyanobacterial densities exceeded 2000 cells/mL.

### 5.4. Quantification of Microcystins in Water Samples

Total microcystins in water samples were analyzed by enzyme-linked immunosorbent-assay (ELISA), using a specific plate kit (Abraxis, polyclonal indirect competitive ADDA ELISA, Warminstair, PA, USA), according to the manufacturer instructions. Previously frozen 5 mL aliquots were defrosted at room temperature (one freeze/thaw cycle) and sonicated (Sonics Vibracell V505 probe, Newtown, CT, USA) for 2 min (10 s sonication/2 s rest cycle) to release intracellular microcystins. Absorbance was measured at 450 nm using a microplate ELISA photometer (Thermo, Labsystems Multiskan Ascent^®^, Helsinki, Finland) and acceptable sample variance was <15%. Results were obtained from two replicate samples and are given in μg MCLR equivalents per L water. This method provides a detection and quantification limit of 0.10 and 0.15 μg/L, respectively.

### 5.5. Risk Levels and Human Exposure Scenarios

The risk levels of each reservoir were calculated according to the WHO Guidelines in bathing waters [26], which comprises the criteria of cyanobacterial cell density and the criteria of microcystin concentration (Table 5). For human exposure scenarios, calculations of minimum water ingestion to reach TDI were performed accordingly to Gkelis et al. [56].

## Figures and Tables

**Table 1:** Frequency of overall cyanobacteria bloom and microcystins detection in each freshwater reservoir.

Reservoir	Samples with Blooms (%)	Samples with Microcystins (%)
A1	30	0
A2	25	0
A3	15	0
A4	44	16
A5	77	24
A6	100	85
A7	69	38
A8	79	58

**Table 2:** Cyanobacterial species, maximum cell density and frequency of occurrence and blooming of each species identified in the studied reservoirs.

Cyanobacteria Species	Maximum Cell Density (Cells/mL)	Frequency of Occurrence (% of Samples)	Frequency of Blooming (% of Samples)
**Reservoir A1**			
*Aphanizomenon* sp.	26	1	0
*Aphanizomenon gracile*	20	1	0
*Aphanothece* sp.	4980	11	4
*Aphanocapsa* sp.	10,314	3	1
*Aphanocapsa holsatica*	410	1	0
*Coelosphaerium* sp.	266	1	0
*Chroococcus* sp.	10	1	0
CHROOCOCCALES n.i.	23,820	26	8
*Dolichospermum* sp.	506	5	0
*Gomphosphaeria* sp.	169	3	0
*Gloeothece linearis*	7426	1	1
*Leptolyngbya* sp.	233	1	0
*Merismopedia* sp.	128	2	0
*Microcystis* sp.	1078	6	0
*Microcystis aeruginosa*	3371	28	2
*Microcystis incerta*	210	1	0
NOSTOCALES n.i.	77	1	0
*Oscillatoria* sp.	3677	2	1
OSCILLATORIALES n.i.	659	3	0
*Planktolyngbya limnetica*	181	2	0
*Pseudanabaena* sp.	4157	9	1
*Pseudanabaena limnetica*	181	3	0
*Synechocystis* sp.	10	1	0
*Woronichinia naegeliana*	572	2	0
**Reservoir A2**			
*Aphanothece* sp.	17,408	5	5
*Aphanocapsa* sp.	20	3	0
CHROOCOCCALES n.i.	5198	30	3
*Lyngbya* sp.	92	3	0
*Microcystis* sp.	352	3	0
*Microcystis aeruginosa*	11,311	23	3
*Microcystis wesenberghii*	218	5	0
*Oscillatoria* sp.	153	5	0
*Pseudanabaena* sp.	3325	10	5
OSCILLATORIALES n.i.	919	10	0
**Reservoir A3**			
*Aphanizomenon* sp.	52	2	0
*Aphanizomenon flos-aquae*	14	2	0
*Aphanothece* sp.	271	2	0
*Aphanocapsa* sp.	2129	4	2
*Aphanocapsa incerta*	233	2	0
*Chroococcus* sp.	20	2	0
CHROOCOCCALES n.i.	4474	18	4
*Dactylococcopsis fascicularis*	31	4	0
*Microcystis* sp.	511	2	0
*Microcystis aeruginosa*	1736	16	0
*Microcystis wesenberghii*	174	2	0
*Merismopedia* sp.	20	2	0
*Planktothrix agardhii*	48	2	0
*Pseudanabaena* sp.	250	5	0
*Pseudanabaena limnetica*	276	9	0
*Phormidium* sp.	215	2	0
*Snowella* sp.	363	2	0
*Snowella atomus*	5576	2	2
*Snowella litoralis*	4624	2	2
**Reservoir A4**			
*Aphanothece* sp.	1098	5	0
*Anathece clathrata*	4876	1	1
*Aphanocapsa* sp.	14,076	1	1
*Aphanocapsa holsatica*	1352	1	0
*Chroococcus* sp.	817	2	0
CHROOCOCCALES n.i.	63,177	25	4
*Coelosphaerium* sp.	270	1	0
*Dactylococcopsis fascicularis*	3146	4	1
*Dolichospermum* sp.	312	4	0
*Gomphosphaeria* sp.	409	2	0
*Leptolyngbya* sp.	306	1	0
*Microcystis* sp.	521	2	0
*Microcystis aeruginosa*	35,301	29	19
*Microcystis ichthyoblabe*	28,990	5	1
*Microcystis wesenberghii*	20,429	1	1
*Merismopedia* sp.	61	3	0
NOSTOCALES n.i.	358	3	0
*Planktolyngbya limnetica*	405	3	0
*Planktothrix* sp.	521	3	0
*Planktothrix agardhii*	526	1	0
*Planktothrix isothrix*	1181	1	0
*Planktothrix pseudoagardhii*	271	1	0
*Oscillatoria* sp.	255	1	0
*Oscillatoria lacustris*	695	1	0
*Oscillatoria lauterbornii*	100	1	0
*Pseudanabaena* sp.	10,010	3	0
*Pseudanabaena limnetica*	171	1	0
*Pseudanabaena catenata*	210	3	0
*Pseudanabaena redekei*	52	1	0
*Phormidium* sp.	1154	3	0
*Snowella* sp.	603	1	0
*Synechocystis* sp.	112	2	0
OSCILLATORIALES n.i.	919	4	0
*Woronichinia naegeliana*	293	1	0
**Reservoir A5**			
*Aphanizomenon* sp.	14,632	10	10
*Aphanizomenon flos-aquae*	144,339	38	33
*Aphanothece* sp.	225	5	0
CHROOCOCCALES n.i.	2197	62	5
*Dactylococcopsis* sp.	82	10	0
*Dolichospermum* sp.	13,829	14	10
*Dolichospermum flos-aquae*	136	5	0
*Gomphosphaeria* sp.	81,165	19	10
*Microcystis* sp.	2370	5	5
*Microcystis aeruginosa*	426,966	43	24
*Microcystis wesenberghii*	791	5	0
*Merismopedia* sp.	198	5	0
NOSTOCALES n.i.	664	14	0
*Oscillatoria* sp.	51,858	10	5
*Pseudanabaena* sp.	1451	5	0
**Reservoir A6**			
*Aphanizomenon* sp.	65,307	8	8
*Aphanizomenon flos-aquae*	319,216	77	69
*Aphanizomenon issatschenkoi*	138,820	54	31
*Aphanothece* sp.	4092	8	8
*Coelosphaerium* sp.	14,405	8	8
CHROOCOCCALES n.i.	9939	31	31
*Dactylococcopsis* sp.	288	15	0
*Dolichospermum* sp.	14,117	23	23
*Dolichospermum aphanizomenoides*	81,648	23	23
*Dolichospermum spiroides*	2017	8	8
*Gomphosphaeria* sp.	11,524	23	23
*Cylindrospermopsis raciborskii*	124,069	15	15
*Microcystis aeruginosa*	169,156	69	54
*Microcystis incerta*	74,741	15	15
*Merismopedia* sp.	2305	23	8
*Oscillatoria* sp.	451,261	77	69
*Trichodesmium* sp.	2017	8	8
OSCILLATORIALES n.i.	60,616	15	8
**Reservoir A7**			
*Anabaenopsis* sp.	32	3	0
*Aphanizomenon* sp.	603	6	0
*Aphanizomenon flos-aquae*	50,153	38	16
*Aphanizomenon flos-aquae* var. *klebahnii*	10,704,801	3	3
*Aphanizomenon yezoense*	251,865	6	6
*Aphanizomenon gracile*	77,835	6	6
*Aphanizomenon issatschenkoi*	47,702	6	6
*Aphanothece* sp.	1596	9	0
*Chroococcus* sp.	613	3	0
CHROOCOCCALES n.i.	5585	47	6
*Dactylococcopsis* sp.	41	9	0
*Dolichospermum* sp.	112,615	16	6
*Dolichospermum lemmermannii*	85,802	3	3
*Dolichospermum mendotae*	1618	3	0
*Dolichospermum solitaria*	378	6	0
*Dolichospermum spiroides*	21,450	3	3
*Gomphosphaeria* sp.	24,686	25	13
*Microcystis* sp.	12,492	13	9
*Microcystis aeruginosa*	116,828	50	19
*Microcystis ichthyoblabe*	1,578,141	9	9
*Merismopedia* sp.	2043	22	3
NOSTOCALES n.i.	377	3	0
*Planktothrix* sp.	162,104	3	3
*Oscillatoria* sp.	1239	9	0
*Pseudanabaena* sp.	6742	9	3
*Raphidiopsis mediterranea*	16,445	3	3
*Spirulina* sp.	34	3	0
OSCILLATORIALES n.i.	807	13	0
*Woronichinia naegeliana*	3,233,912	9	9
**Reservoir A8**			
*Anabaenopsis* sp.	39,632	2	2
*Aphanizomenon* sp.	1,297,190	11	9
*Aphanizomenon flos-aquae*	2,027,579	42	38
*Aphanizomenon flos-aquae* var. *klebahnii*	3,282,942	2	2
*Aphanizomenon gracile*	129,888	15	15
*Aphanizomenon issatschenkoi*	959,142	26	25
*Aphanizomenon aphanizomenoides*	203,146	2	2
*Aphanothece* sp.	11,747	6	6
CHROOCOCCALES n.i.	98,876	25	11
*Dactylococcopsis* sp.	6946	2	2
*Dolichospermum* sp.	2,166,292	43	34
*Dolichospermum aphanizomenoides*	413,483	4	2
*Dolichospermum circinalis*	9193	8	8
*Dolichospermum flos-aquae*	652,482	4	4
*Dolichospermum solitaria*	225	2	0
*Dolichospermum spiroides*	22,483	8	8
*Microcystis incerta*	53,236	6	6
*Gomphosphaeria* sp.	2,200,096	28	25
*Merismopedia* sp.	4787	6	2
*Planktothrix* sp.	233,606	13	13
*Planktothrix agardhii*	612,870	2	2
*Planktothrix clathrata*	194,076	2	2
*Planktothrix isothrix*	426,966	4	4
*Oscillatoria* sp.	985,618	25	23
*Pseudanabaena* sp.	1788	4	0
*Pseudanabaena mucicola*	140,960	4	2
*Phormidium* sp.	94,995	2	2
*Raphidiopsis* sp.	2247	2	2
*Raphidiopsis mediterranea*	26,558	2	2
OSCILLATORIALES n.i.	130,950	11	6
*Woronichinia naegeliana*	1,395,301	8	8

**Table 3:** Scenarios of human exposure to microcystins in the studied reservoirs, considering the tolerable daily intake (TDI).

Reservoirs	MC Range Detected (µg/L)	Minimum Water Ingestion to Reach TDI (mL)	
		Children	Adults
A1	-	-	-
A2	-	-	-
A3	-	-	-
A4	0.16–2	201	1206
A5	0.37–3.7	108	647
A6	0.1–4.1	98	585
A7	0.16–506	1	5
A8	0.2–389	1	6

**Table 4:** Characteristics of the sampled freshwater reservoirs [53,54], monitoring period and number of collected samples.

Reservoirs	Total Capacity (dam^3^)	Surface Area (ha)	Mean Depth (m)	Other Uses	Trophic State	Years of Monitoring	Number of Samples
A1	72,000	2023	35.6	Energy production	Mesotrophic	14	109
A2	1,095,000	3291	33.3	Drinking water supply	Oligotrophic	10	40
				Energy production			
				Flood defense			
A3	53,700	246	21.8	Energy production	Mesotrophic	9	56
A4	660	12	6.4	Drinking water supply	Mesotrophic	14	112
				Flood defense			
A5	ND	ND	ND	ND	ND	3	21
A6	ND	ND	ND	ND	ND	2	13
A7	ND	ND	ND	ND	ND	8	32
A8	3032	124	3.8	Irrigation	Eutrophic	11	53

ND—Not described.

**Table 5:** Criteria used to determine the risk levels in the freshwater reservoirs, based on the WHO Guidelines for bathing waters [26].

Risk Level	Criteria Based on Cyanobacterial Density (Cells/mL)	Criteria Based on Microcystin Concentration (µg MC/L)
No Risk	Not detected	Not detected or not analysed *
Low Risk	<20,000	<20
Moderate Risk	20,000–100,000	-
High Risk	>100,000	≥20

* When the density of potential toxic cyanobacteria was bellow 2000 cells/mL.
